# Monitoring of Cerebral Blood Flow Autoregulation after Cardiac Arrest

**DOI:** 10.3390/medicina60091381

**Published:** 2024-08-23

**Authors:** Rok Petrovčič, Martin Rakusa, Andrej Markota

**Affiliations:** 1 Emergency Department, University Medical Centre Maribor, Ljubljanska ulica 5, 2000 Maribor, Slovenia; 2 Department of Neurologic Diseases, University Medical Centre Maribor, Ljubljanska ulica 5, 2000 Maribor, Slovenia; ris101@gmail.com; 3 Department of Intensive Internal Medicine, Division of Internal Medicine, University Medical Centre Maribor, Ljubljanska ulica 5, 2000 Maribor, Slovenia; andrej.markota@ukc-mb.si

**Keywords:** cardiac arrest, cerebral blood flow, autoregulation, monitoring, post-cardiac arrest syndrome

## Abstract

*Background:* Cardiac arrest remains one of the leading causes of death. After successful resuscitation of patients in cardiac arrest, post-cardiac arrest syndrome develops, part of it being an impaired cerebral blood flow autoregulation. Monitoring cerebral blood flow autoregulation after cardiac arrest is important for optimizing patient care and prognosticating patients’ survival, yet remains a challenge. There are still gaps in clinical implications and everyday use. In this article, we present a systematic review of studies with different methods of monitoring cerebral blood flow autoregulation after non-traumatic cardiac arrest. *Methods*: A comprehensive literature search was performed from 1 June 2024 to 27 June 2024 by using multiple databases: PubMed, Web of Science, and the Cochrane Central Register of Controlled Trials. Inclusion criteria were studies with an included description of the measurement of cerebral blood flow autoregulation in adult patients after non-traumatic cardiac arrest. *Results:* A total of 16 studies met inclusion criteria. Our data show that the most used methods in the reviewed studies were near-infrared spectroscopy and transcranial Doppler. The most used mathematical methods for calculating cerebral autoregulation were cerebral oximetry index, tissue oxygenation reactivity index, and mean flow index. *Conclusions:* The use of various monitoring and mathematical methods for calculating cerebral blood flow autoregulation poses a challenge for standardization, validation, and daily use in clinical practice. In the future studies, focus should be considered on clinical validation and transitioning autoregulation monitoring techniques to everyday clinical practice, which could improve the survival outcomes of patients after cardiac arrest.

## 1. Introduction

Cardiac arrest is one of the leading causes of death in Europe [1,2,3]. Appropriate treatment after cardiac arrest consists of immediate basic life support, followed by advanced life support [4]. The chain of survival is important, connecting basic and advanced life support with post-resuscitation care [5]. Part of post-cardiac arrest syndrome is post-cardiac arrest brain injury—hypoxic brain injury, due to impaired cerebral blood flow autoregulation [6].

Cerebral blood flow autoregulation (CBFA) is a “*homeostatic process that regulates and maintains cerebral blood flow constantly across a range of blood pressures*” [7]. Niels Lassen proposed the original concept in 1959 with a figure of a lower limit and the plateau of mean arterial pressure in which cerebral blood flow remains constant [8]. Through the years and with the advances in monitoring techniques, the concept has been challenged in terms of the smaller cerebral antiregulatory plateau and CBFA being more pressure-passive. To this day, there are important knowledge gaps in understanding CBFA [9,10]. Assessment of CBFA can be divided into static and dynamic. The static method assesses relationships between CBFA and mean arterial pressure (MAP) when they reach a steady state without time changes. The dynamic method assesses changes in CBFA in response to dynamic changes in MAP [7,10].

In clinical practice, CBFA is monitored in settings of patients after cardiac arrest, traumatic brain injury, brain hemorrhage, stroke, during anesthesia, sepsis, and other critically ill patients [11]. The most commonly used techniques include near-infrared spectroscopy (NIRS), transcranial Doppler ultrasound (TCD), intracranial pressure, and diffuse correlation spectroscopy (DCA) [12]. To current date, none of the monitoring techniques are defined as a golden standard, and a lack of implication in clinical management protocols exists [13].

Mathematical methods used for calculation of CBFA.

There is a large variety of mathematical methods or indices used to calculate and define impaired CBFA by data derived from monitoring techniques [12]. Some of the frequently used mathematical methods include [14] the following.

Autoregulation index (ARI) uses arterial blood pressure (ABP) and blood flow velocity (Fv) as input signals. The signals are interpreted as an ARI value of 0 (meaning absent autoregulation) and an ARI value of 9 (meaning normal autoregulation) [15,16].

The flow index uses ABP (as cerebral perfusion pressure) and Fv as an input signal. The signals are interpreted as higher values of mean flow index (Mx), systolic flow index (Sx), or diastolic flow index (Dx) means impaired autoregulation [16,17,18].

The transfer function uses ABP and Fv as input signals. It uses Fourier decomposition of stationary input and output signals and phase, gain, and coherence. Impaired autoregulation is defined as low phase, high gain, and high coherence [19].

The cerebral oximetry index (COx) and tissue oxygenation reactivity index (TOx) use ABP (as cerebral perfusion pressure) and NIRS-measured brain oxygenation as input signals. It is calculated as a Pearson correlation coefficient between 30 consecutive 10-s means of ABP and tissue oxygenation. Higher values of COx or TOx mean impaired autoregulation [20,21,22,23].

The pressure reactivity index (PRx) uses ABP and ICP as input signals. It is calculated as a moving correlation of 30 consecutive 10-s means of ABP and ICP. Higher values of PRx mean impaired autoregulation [24].

The oxygen reactivity index (ORx) uses cerebral perfusion pressure and brain tissue oxygen (PBTO2) pressure as the input signal. It is calculated as a correlation between 30 consecutive 10-s means of ABP and PBTO2. Higher values of ORx mean impaired autoregulation [25,26].

Monitoring CBFA in patients after cardiac arrest has clinical implications regarding prognostication of cerebral performance and tailoring patients’ cerebral perfusion pressure [27].

This systematic review aims to identify and list monitoring techniques and mathematical methods used in clinical trials to calculate and define impaired cerebral blood flow autoregulation in patients after non-traumatic cardiac arrest.

## 2. Materials and Methods

### 2.1. Data Sources and Search Strategy

**Figure 1:**
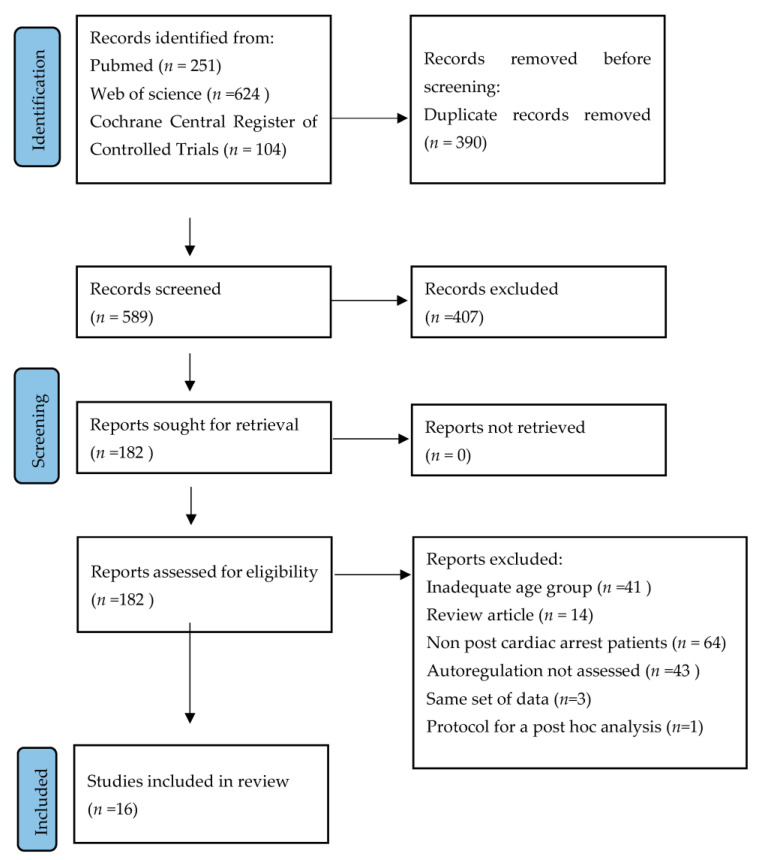
PRISMA literature review flow diagram.

In this study, we used Preferred Reporting Items for Systematic Review and Meta-Analysis Protocols (PRISMA-P) guidelines (Figure 1) [28]. A systematic literature search was performed from 1 June 2024 to 27 June 2024 by using the following databases to identify relevant studies: PubMed, Web of Science, and the Cochrane Central Register of Controlled Trials. The following search terms were used: monitoring, cardiac arrest, cerebral, blood flow, and autoregulation, with the following derivatives and combinations: (“Monitoring” OR “Measurement” OR “Assessment” OR “Evaluation”) AND (“Cerebral Blood Flow” OR “Cerebral Perfusion” OR “Brain Blood Flow”) AND (“Cardiac Arrest” OR “Heart Arrest” OR “Cardiopulmonary Arrest”); (“Cerebral Blood Flow” OR “Cerebral Perfusion” OR “Brain Blood Flow”) AND (“Autoregulation” OR “Self-Regulation” OR “Cerebral Autoregulation”) AND (“Cardiac Arrest” OR “Heart Arrest” OR “Cardiopulmonary Arrest”); and (“Monitoring” OR “Measurement” OR “Assessment” OR “Evaluation”) AND (“Autoregulation” OR “Self-Regulation” OR “Cerebral Autoregulation”) AND (“Cardiac Arrest” OR “Heart Arrest” OR “Cardiopulmonary Arrest”). The following selected filters were used: humans, language (English), and time of publication (from 1 January 1990). Inclusion criteria were studies with an included description of the measurement of cerebral blood flow autoregulation in adult patients after non-traumatic cardiac arrest. The exclusion criteria were adolescents (under 18 years of age) and traumatic cardiac arrest. Searches were re-run and updated before the final analyses.

### 2.2. Study Selection and Data Collection

Two authors (RP and AM) independently searched articles, screening titles, abstracts, and full texts. The articles were categorized into three groups: “included” and “excluded” (if both examiners agree) or “uncertain” (in case of disagreement). If the article was classified as uncertain, the decision was made by future examination of a third author (MR). A standardized electronic spreadsheet (Microsoft Excel, version 2016; Microsoft, Redmond, WA, USA) was used to extract data. Data were synthesized in a standardized electronic spreadsheet, with columns including the following titles: study, main finding, type of study, setting, patients (number), monitoring method, maneuver used (if applicable), main limitation, and autoregulation indices (mathematical method used) (Table 1).

### 2.3. Endpoints

Representation of monitoring techniques and mathematical methods used to calculate and define impaired cerebral blood flow autoregulation in patients after non-traumatic cardiac arrest.

Risk of Bias Assessment in the Included Studies.

Two examiners (RP and AM) independently assessed the risk of bias in the included studies using a Mixed Methods Appraisal Tool (MMAT), version 2018.

## 3. Results

The Prisma literature review flow diagram is presented in Figure 1. We identified 182 studies, of which 16 were analyzed (Table 1). Three studies were excluded as they used the same data set as in already included studies. One was a protocol for post hoc analyses. In total, 13 studies were prospective and 3 were retrospective. Altogether, 531 patients were included.

In the analyzed studies, a large variety of methods and mathematical models were used to assess cerebral autoregulation (Table 2 and Table 3). The most commonly used methods were NIRS and TCD. Other methods were also used. In total, 3 out of 16 studies used invasive monitoring methods. CBFA was calculated using data derived from these methods and autoregulation metrics.

Several mathematical methods or autoregulation metrics were described for the calculation of CBFA in the included studies. The most commonly used methods were COx, Tox, and Mx. They correlate well with the mostly used monitoring methods in studies—NIRS and TCD. In the majority of studies, blood pressure was monitored invasively through an arterial line.

## 4. Discussion

Our analysis demonstrates heterogeneity in assessing CBFA. Several different monitoring techniques and outcome indices (COx, PRx, TOx, and Mx) were used to better understand the brain’s autoregulatory function. While each study design contributes unique insights, the heterogeneity poses challenges when attempting to synthesize and draw overarching conclusions.

Heterogeneity in Monitoring Approaches

The most used method of CBFA measurement in CA patients is NIRS. The strength of this method is the noninvasive real-time monitoring of brain oxygenation based on the principle of transluminal spectroscopy. Despite some of its limitations, for example, the limitation of signal capture only to the area of the frontal cortex and the oxygenation value changes, as a result of the changes in blood flow in the skin, NIRS is adopted in various clinical practices [45]. Because of its strengths, it is widely used to monitor autoregulation in neonatology, surgery, and neurocritical care [45]. In most analyzed studies, data derived from NIRS are analyzed using ICM+ ^®^ software in the form of COx. It also is used in a continuous assessment of CBFA in adults [22].

The second most used method is TCD, a noninvasive real-time monitoring method of cerebral blood flow velocity in the main intracranial vessels [46]. TCD is a valid method for determining the lower limit of cerebral autoregulation [47]. TCD has been used to monitor autoregulation in different settings, similar to NIRS, and also to evaluate patients with postpartum angiopathy, eclampsia, and syncope [48]. Limitations of its use are being operator-dependent, its reproducibility, and its variability [49].

Multimodal monitoring of CBFA can provide additional insight into complex interrelated neurophysiologic determinants of CBFA but can be challenging in terms of cost and clinical efficiency [50].

Association with Clinical Outcomes

Several studies demonstrated associations between impaired CBFA and clinical outcomes in CA patients. Ameloot et al. reported that CBFA was disturbed in one-third of CA patients. The survival rate was negatively associated with the time spent under optimal mean arterial pressure (MAP) [29]. Similarly, Pham et al. reported that impaired CBFA was independently associated with mortality at three months follow-up [34]. In another study, Laurikkala et al. reported disturbances in CBFA in a significant proportion of CA patients, which were correlated with worse outcomes [36]. Therefore, we may conclude that monitoring CBFA in this population is important and may help predict survival outcomes. Monitoring of CBFA can also be used as a prediction tool in other cerebrovascular pathologies [51,52,53,54]. There is still a lack of definite evidence, which makes everyday use of CBFA as a prediction tool for clinical outcomes difficult [55].

However, it is necessary to use a reliable and robust method for CBFA monitoring. One possibility is NIRS. When comparing left- and right-sided NIRS recordings, there were no differences between sides or CBFA estimation in CA patients, indicating the reliability of NIRS as a monitoring tool [40]. Monitoring CBFA with NIRS-derived COx correlated well with previously validated TCD-based methods, suggesting the reliability of NIRS in assessing CBFA [42].

In research on pediatric patients resuscitated after cardiac arrest, monitoring of CBFA is often used. Similarly, there is a high heterogeneity of methods in adult patients after cardiac arrest, which includes NIRS, ICP, and TCD, with a variety of mathematical methods used [56,57,58,59,60].

Autoregulation after cardiac arrest is experimentally explored in swine models using NIRS, ICP, and laser-Doppler flowmetry [61,62,63].

Different pathologies cause cerebral dysfunction and CBFA is monitored in other clinical settings. In sepsis, cerebral autoregulation is mostly monitored using NIRS and TCD [64,65,66]. In subarachnoid hemorrhage, cerebral autoregulation is monitored similarly and uses data derived from ICP [26,67,68,69,70]. In cardiac surgery, autoregulation is widely monitored with the same techniques [71,72,73].

Other systematic reviews found similar conclusions. In the review of determining optimal mean arterial pressure after cardiac arrest by Rikhraj et al., a high heterogeneity of monitoring methods and calculation was found [74]. Longhitano et. al. reviewed CBFA in non-brain injured patients. NIRS and TCD assessed CBFA in the included studies. When NIRS was used, cerebral autoregulation was monitored using Pearson’s correlation coefficient between MAP and NIRS signals to generate the variable COx. When TCD was used, the Mx index was calculated to evaluate cerebral autoregulation as a correlation between systemic MAP and mean flow velocity in the middle cerebral artery [75]. A review by Caldas et al. of CBFA in cardiopulmonary bypass surgery found that cerebral autoregulation was mostly assessed by NIRS and TCD with a wide variety of indices of static and dynamic autoregulation [76]. A similarity was found in the assessment of the risk of perioperative stroke and CBFA monitoring by Santos et al. [77]. By the analysis of predictors of outcome with cerebral autoregulation monitoring by Rivera-Lara et al., invasive (ICP monitor) and noninvasive (NIRS, TCD) methods were identified to monitor cerebral autoregulation in different pathologies, with different autoregulation indices—the PRx, ARI, ORx, and flow reactivity index [78]. In a systematic review and meta-analysis by De-Lima-Oliveira, with the purpose of establishing the relation between cerebral autoregulation and intracranial hypertension, identified techniques were divided into static and dynamic. Static techniques of cerebral autoregulation monitoring identified in studies were Xe133, arterio-jugular differences in oxygen, positron emission tomography flow, and N2O. Dynamic techniques were used to move the correlation index to assess CA. The techniques to determine the correlations with blood pressure variation were invasive intracranial pressure (PRx), TCD (Mx), cerebral tissue PtbO2 (ORx), and CBF measurements through a laser catheter (Lx) or a thermal sensor catheter (CBFx) [79].

Strengths and Limitations

The strength of this study is the systematic review of autoregulation monitoring techniques employed in studies that involve patients who have experienced non-traumatic cardiac arrest. We were able to identify different monitoring methods and mathematical methods used to measure cerebral autoregulation.

The limitations of this study are the high heterogeneity of different monitoring, mathematical methods, and methodological limitations observed, which makes comparison and reproducibility difficult. There is a small overall size of patient cohorts.

Future research should focus on finding noninvasive reproducible monitoring methods of cerebral blood autoregulation, comparing different metrics, and transitioning the monitoring of cerebral autoregulation to everyday clinical practice. There is still an important gap in the everyday standard clinical use of cerebral autoregulation monitoring in patients after cardiac arrest, which can be addressed with the findings of additional studies.

## 5. Conclusions

In the presenting article, we systematically reviewed different methods of monitoring cerebral blood flow autoregulation after non-traumatic cardiac arrest. The use of diverse monitoring methods and definitions introduces challenges in standardization. The choice of modality may influence the observed outcomes and make comparisons between studies difficult.

Our results demonstrate that the most used methods were NIRS and TCD, with a wide variety of mathematical methods for calculating cerebral autoregulation. The most common were COx, Tox, and Mx.

Future studies should focus on clinical validation and transitioning those methods to everyday clinical practice to improve the survival outcomes of patients after CA.

## Figures and Tables

**Table 1:** Studies analyzed.

Study	Type of Study	Main Finding	Setting	Study Population	Monitoring Method	Maneuver	Main Limitation	Autoregulation Indices
Ameloot K et al. [29]	Prospective observational study	CBFA disturbed in one-third CA patients, correlated with worse outcomes. Time spent under optimal MAP is negatively associated with survival	Tertiary care hospital	51 patients after CA	NIRS, invasive blood pressure monitoring	/	In a small sample size, COX predicted optimal MAP as an average value, insufficient data on drugs used	COx
Nishizawa H et al. [30]	Prospective interventional study	CBFA impaired in patients after CA	Department of Anesthesiology, University school of medicine	Eight patients after CA	A catheter was inserted percutaneously into the right internal jugular vein, the tip positioned in the jugular bulb for venous blood gas measurement. Arterial pressure measurement and arterial blood gas samples were obtained	MAP changed to a value of 30% lower or higher than baseline by infusing trimethaphan or methoxamine	Small sample size	Arterial-jugular bulb venous oxygen content difference (AVDO2) calculated at each MAP level. After that, 1/AVDO2, cerebral blood flow indices (CBFI) were calculated. The changes in CBFI and oxygen saturation of jugular venous blood seen after the decrease or increase in MAP indicates impairment of CBFA
Sundgreen C et al. [31]	Case-control interventional study	CBFA is absent or right-shifted in the majority of patients after CA	University Hospital ICU	18 patients after CA in and out of hospital, 6 healthy control subjects	TCD, invasive blood pressure monitoring	A stepwise rise in MAP by use of norepinephrine infusion	Small sample size	MAP was plotted against the mean Fv, and a possible lower limit of autoregulation was identified
Sekhon MS et al. [32]	Prospective interventional study	A linear relationship between increased MAP and PBTO2 in HIBI patients. Perfusion within proximity of optimal MAP may be associated with improved PBTO2	Quaternary ICU	Ten patients after CA	Multimodal: brain tissue oxygenation, intracranial pressure, jugular venous continuous oximetry, NIRS	/	Small sample size, definition of brain hypoxia from the traumatic brain injury literature, patient cohort with pulseless circulatory arrest	PRx
Sekhon MS et al. [33]	Proof-of-concept feasibility study	Feasibility of determining optimal MAP using cerebral oximetry in patients after CA	General hospital ICU	20 patients after CA	NIRS, invasive blood pressure monitoring	/	Proof of concept study, no granular data on carbon dioxide	COx
Pham P et al. [34]	Case-control study	Early impairment of CBFA after CA independently associated with mortality at three months follow-ups	General hospital ICU	23 patients after CA, 28 healthy volunteers	NIRS, invasive blood pressure monitoring, fino-meter for noninvasive blood pressure monitoring in healthy volunteers	Blood pressure changes induced in healthy volunteers through bed-tilt positional changes, Valsalva maneuvers and short immersion of one hand in ice-cold water	Small sample size, intermittent measurement	TOx
Van den Brule JMD et al. [35]	Prospective observational study	Changes in experimental settings strongly influence the results of estimation of CBFA	University Hospital ICU	13 patients after CA	TCD, invasive blood pressure monitoring	Repeated changes in the position of the bed from horizontal to a maximum of 30 degrees Trendelenburg and 30 degrees anti-Trendelenburg	Small sample size, changes in quality of the recorded signal, the influence of bed position on venous outflow	CBFA was calculated in the time domain (Mx) and frequency domain (transfer function analysis)
Laurikkala J et al. [36]	Post-hoc analysis of multicenter srandomised controlled pilot study	Impaired cerebrovascular reactivity is common after CA, especially in patients with chronic hypertension. Decreased upper MAP bound and a narrower MAP range for maintained cerebrovascular reactivity associated with poor outcome, severe brain injury assessed by NfL	Six ICUs in Finland and one ICU in Denmark	120 patients after CA	NIRS, invasive blood pressure monitoring	/	Limitations of NIRS and TOx to monitor cerebrovascular reactivity	TOx
Bindra J et al. [37]	Prospective observational study	Dynamic CBFA can be continuously assessed noninvasively	General hospital ICU	Diverse cohort, including 1 patient after CA	NIRS, invasive blood pressure monitoring, finometer for noninvasive blood pressure monitoring	/	Small sample size, finometer being operator depended	CBFA as a correlation coefficient between invasive arterial blood pressure and rSO2 (iTOx) or noninvasive arterial blood pressure and rSO2 (nTOx)
Calviello LA et al. [38]	Prospective study	Multiparameter TCD neuromonitoring increases outcome predictive power. TCD-based indices can be applied to general intensive care monitoring	University Hospital ICU	Diverse cohort, including 14 patients after CA	TCD and ABP were continuously monitored non-invasively using a finger probe or a pressure monitoring kit	/	Small sample size, preliminary results, patients not separated by condition	Mx
Balu R et al. [39]	Retrospective observational study	Cerebrovascular pressure reactivity and ICP appear to be associated with neurologic outcomes in patients with HIBI	NCCU at University Hospital	Diverse cohort, including 32 patients after CA	ICP monitoring and interstitial brain tissue oxygen measurements through quad-lumen bolt invasive blood pressure monitoring	/	Single-center retrospective design, selection and indication biases, monitoring only the frontal lobe, temporal trends not analyzed, not using cerebral performance score as an outcome	PRx
Hazenberg L et al. [40]	Prospective observational study	No differences between left and right-sided NIRS recordings or CBFA estimation in HIBI patients	ICU University Medical Center	11 patients after CA	NIRS, invasive blood pressure monitoring	/	Small sample size, short monitoring time, possibility of local pathology	COx
Griesdale DEG et al. [41]	Prospective multicenter cohort study	It is feasible to recruit and collect high-frequency physiological data in patients after CA. Time below optimal MAP and duration of dysfunctional CBFA not associated with an unfavorable neurologic outcome	ICUs in three teaching hospitals	59 patients after CA	NIRS, invasive blood pressure monitoring	/	Underpowered analyses, inability to control for potential confounds	COx
Rivera-Lara L et al. [42]	Prospective observational cohort study	Monitoring CBFA with NIRS-derived COx is correlated and agrees well with previously validated TCD-based methods	NCCU at the teaching hospital	Diverse cohort, including one patient after CA	NIRS, TCD, invasive blood pressure monitoring	/	Small sample size, heterogeneous lesions in a population with acute coma	COx, Mx
Crippa IA et al. [43]	Retrospective analysis of prospectively collected data	CBFA is frequently altered in CA patients treated by targeted temperature management. Altered CBFA during normothermia was independently associated with poor outcome	Department of intensive care at University hospital	50 patients after CA	TCD, invasive blood pressure monitoring	/	Cerebrovascular resistance or absolute CBF values were not measured directly MAP in relation to individual CBFA curves not investigated	Mx
Tachino J et al. [44]	A prospective, observational cohort study	Mortality increased significantly with longer non-CBFA time within 96 h after the return of spontaneous circulation	Trauma and Acute Critical Care Center	100 consecutive patients after CA	NIRS, invasive blood pressure monitoring	/	Observational research and differences in patient characteristics and clinical protocol did not account for the potential influence of targeted temperature control and CO_2_ on cerebral circulation	COx

Abbreviations: NCCU: Neurocritical care unit, ICU: Intensive care unit, CA: cardiac arrest, NIRS: near-infrared spectroscopy, TCD: transcranial Doppler, ICP: intracranial pressure, ABP: arterial blood pressure, MAP: mean arterial pressure, CBFA: Cerebral blood flow autoregulation, rSO2: regional saturation of oxygen, Fv: blood flow velocity, Mx: mean flow index, COx: cerebral oximetry index, TOx: tissue oxygenation reactivity index, PRx: Pressure reactivity index, PBTO2: pressure of brain tissue oxygen, HIBI: hypoxic-ischemic brain injury, NfL: neurofilament light chain.

**Table 2:** Monitoring methods used in research.

Monitoring Method	Number of Research
NIRS	8
TCD	4
ICP and interstitial brain tissue oxygen measurements through quad-lumen bolt	1
Internal jugular vein catheter	1
Multimodal	2

Abbreviations: NIRS: near-infrared spectroscopy, TCD: transcranial Doppler, ICP: intracranial pressure.

**Table 3:** Mathematical models used in research.

Mathematical Model	Number of Research
COx	5
TOx	3
Mx	3
PRx	2
Multimodal	1
Other	2

Abbreviations: COx: cerebral oximetry index, TOx: tissue oxygenation reactivity index, Mx: mean flow index, PRx—pressure reactivity index.

## Data Availability

All data generated or analyzed during this study are included in this published article.
